# Early stages of learning in interprofessional education: stepping towards collective competence for healthcare teams

**DOI:** 10.1186/s12909-023-04665-8

**Published:** 2023-09-22

**Authors:** Sandra Kemp, Margo Brewer

**Affiliations:** 1 Graduate School of Medicine, University of Wollongong, Wollongong, Australia; 2 Curtin School of Allied Health, Curtin University, Perth, Australia

**Keywords:** Interprofessional education, Interprofessional teams, Curriculum design, Graphic elicitation, Interprofessional collaboration

## Abstract

**Background:**

Interprofessional education (IPE) is a core element of many health professional education curricula. To date the focus of much research has been on student perceptions of, and attitudes towards, the learning experience. Little is known about the impact of early IPE experience on how students understand and learn about effective interprofessional teamwork.

**Methods:**

This qualitative study involved first year university students enrolled in health professions degrees and investigated their descriptions of interprofessional teamwork through graphic elicitation and interviews. Participants were enrolled in a large-scale interprofessional unit (subject) in the university.

**Results:**

The data were analysed through the lens of a tool that classifies dimensions of interprofessional activity. The findings indicated the majority of students had what was classified as a Stage 1 (or ‘nascent’) understanding of integration between work practices and a Stage 2 (or ‘emerging’) understanding of the dimensions of interprofessional teamwork which were commitment, identity, goals, roles and responsibilities, and interdependence.

**Conclusions:**

Based on the findings, the stages for a learning trajectory for interprofessional education are proposed and each stage is mapped to dimensions of interprofessional activity. A number of pedagogical strategies are suggested in order to move students through this two-stage model of learning and ensure their readiness for interprofessional teamwork as health professionals.

**Supplementary Information:**

The online version contains supplementary material available at 10.1186/s12909-023-04665-8.

## Introduction

The importance of interprofessional education (IPE) for pre-registration healthcare students is widely accepted with many examples of IPE experiences described in the literature. Case studies of teaching [1] and simulation of interprofessional teams [2] are common. However, few studies examine how students learn about interprofessional teamwork at the beginning of pre-registration health professional courses, prior to the development of clinical knowledge and skills related to a profession. A recent scoping review highlighted the lack of preparation for students before placing them in interprofessional teams [3]. Where preparation was provided it was done individually rather than in teams [3].

Interprofessional education for first year students enrolled in different healthcare university courses holds curricula design challenges. Students are new to the university experience and professional identity [4]. Students from different healthcare courses are diverse in their career goals, represent a range of prior academic attainment, and have a variety of understandings about other healthcare professions. In this context, health professions students are required to ultimately develop “collective competence” to work in interprofessional teams [5].

The longitudinal learning trajectory of skills needed for successful interprofessional teamwork has seldom been explored and guidance for curriculum designers is scant. The challenges for designing curricula, progression of skill development, and assessing stages of learning are recognized [6]. Little is known about early IPE encounters in health professions courses such medicine, nursing, physiotherapy, and psychology, beyond student attitudes which have been the focus of much of the research to date [7]. Studies have indicated positive benefits of interprofessional education, in terms of a range of outcomes from changes to perceptions, knowledge and skills, behaviours as well as patient care [7], but it is challenging to determine how much is possible in the early stages of university courses.

Understanding what students learn about interprofessional teamwork from early IPE experiences can clarify stages of student learning. This can provide a springboard to effective curricula design.

## Background

This study investigated student learning about interprofessional teamwork during their first exposure to IPE, at the beginning of their university courses. Participants were first year students in a unit (subject or paper) taken by all students from different health professional courses in the university. The unit represented one quarter of a full-time study load, for one semester. The learning experiences aimed to broadly align with the definition of IPE where “members of two or more professions learn with, from and about each other to improve collaboration and the quality of care and services” [8]. However, at the initial start of a course students typically learn about patients in paper-based activities as authentic or simulated patient encounters are not possible. Therefore, at this early stage of tertiary education in the study context, definitions of ‘multiprofessional education’ are also applicable as two or more professions were learning side by side [9].

The IPE activities were expected to support students enrolled in different courses (leading to a health professional qualification) to ensure opportunities to learn about, from and with each other. Early engagement by students in interprofessional education is desirable as preparation for learning the complexity of interprofessional teamwork.

To date, much of the work in IPE focuses on student learning towards the end of health professional courses as students enter clinical placements and move closer to graduation as a health professional. However, our focus was on learning at the start of university courses to prepare health professionals. Our aim was to develop a fuller understanding of student learning trajectories for effective interprofessional teamwork, particularly in relation to ‘collective competence’ [5], an essential component of quality healthcare. For the theoretical framework underpinning the study, we used the interprofessional classification model [10] which posits that there are six dimensions of effective interprofessional teamwork (shared commitment, shared identity, clear team goals, clear roles and responsibilities, interdependence between team members, and integration between work practices). We used this model as the basis of the end point of learning to be an effective member of an interprofessional team, and our study was designed to better understand how students might be developing skills related to the six dimensions in the model.

Our research questions were:

- How do health professions students understand successful interprofessional teamwork in initial encounters with IPE?
- How could these understandings link to the goal of successful interprofessional teamwork in healthcare?

## Methods

The research methods used to generate data were a combination of a visual method and a qualitative method: (1) participant-led graphic-elicitation (which refers to participants being asked to do a drawing on a specific topic) and (2) one-to-one interviews (which immediately followed the drawing completion) in which the participant’s own drawing was used as stimulus.

Each participant was asked to draw a picture to show a group of different health professionals working together successfully as a team in a healthcare situation. They were asked to include the people and aspects that are important for successful teamwork. Participants were aware that being skilled at drawing was not required, that simple elements such as stick figures were acceptable, and text could be included in the drawing [11]. Each participant was given 20 min to complete the drawing.

Next, the interviewer and participant completed a one-hour interview, using the drawing and a semi-structured interview protocol, with questions about different components of the drawing. Thus, the drawing was used as an aide to discussion of the interview questions and as a stimulus for thinking by the participant [12]. The aim was to explore individual meaning about interprofessional teams that might not be possible using typical interview methods. These rich data facilitate participant reflection on the complexity of situations and enables the researchers to gain insights into hidden aspects of the topic of inquiry [13] as graphic elicitation allows the person to go beyond a verbal mode of thinking to include wider dimensions of experience [14]. Furthermore, cross-comparative analysis across the two data sets (graphics and interviews) allows identification of commonalities and contradictions in evidence [15]. Data from both data sets were included in the final analysis.

### Data collection

All participants were enrolled in an undergraduate first year credit-bearing unit for all students enrolled in a health professional course at a large Australian university. Students learnt foundational knowledge with a focus on academic integrity, professional ethics, the Australian and global healthcare systems, and interprofessional competencies as outlined in Brewer and Jones [16]. Learning outcomes for the unit included understanding roles of other health professionals, fundamentals of working in teams and interprofessional care of a range of patient-cases. This unit was the first encounter with interprofessional education for students, as part of their chosen profession. The 17 students who volunteered to participate in the study were from a range of different health professions courses, including nursing, psychology, pharmacy, and medicine (Table 1). Voucher incentives were offered for participation. All interviews were audio-taped and transcribed verbatim. All drawings were scanned and stored in PDF format.

**Table 1:** Participant enrolment

Number of Participants	Enrolled Course	Participant Identifier Number
6	Psychology	301, 461, 609, 682, 832, 923
2	Human Biology	188, 565
2	Medicine	313, 462
2	Psychology and Commerce	205, 855
1	Laboratory medicine	285
1	Physiotherapy	321
1	Psychology & Marketing	683
1	Nursing	464
1	Molecular Genetics and Biotechnology	790

### Data analysis

The researchers conducted a six-step thematic analysis [17] of all transcript and graphic data sets using both a deductive and inductive approach. Deductive analysis allowed the testing of an explanatory model from pre-existing theory [18]. The explanatory model employed the interprofessional classification model developed by Xyrichis and colleagues [10] which consists of six dimensions for classifying interprofessional teamwork interventions and activities: shared commitment, shared identity, clear team goals, clear roles and responsibilities, interdependence between team members, and integration between work practices. Xyrichis et al. [10] posit that the dimensions represent what happens in effective interprofessional practice. In short, this model provides a rich description, across different dimensions, of the goal of interprofessional education: high-functioning interprofessional teams. The dimensions of the framework were used as a lens to analyse how students understood interprofessional team functioning. Building on this deductive analysis, inductive analysis allowed for a holistic understanding of participant interview data and graphic data and thus ensured all important aspects of the data were captured [14].

The authors began by acknowledging their professional backgrounds as educational experts from different disciplines (medical education and allied health education), regularly reflecting on how these backgrounds may have influenced our interpretation of the data [17]. After familiarising ourselves with the entire data set, five randomly selected transcripts and paired graphics were independently analysed in relation the interprofessional activity framework [10]. Commonalities and contradictions between the transcript and graphic were explored. The initial codes were revised until consensus was met, then applied to the remaining data through an iterative process, involving approximately ten hours of discussion until consensus was reached on the coding of the data. The final stage of coding was an inductive process where the entire data set was re-examined. Through this process different stages of learning emerged. The codes were organised into themes and the stages of learning finalised. The full data set was reviewed in relation to these themes and stages, and representative quotations and graphics compiled.

### Ethical considerations

Institutional Review Board approval (Curtin University) for the project was obtained (HRE2018-0020).

## Results

From the deductive analysis phase, all six dimensions from the interprofessional classification model [10] were evident in participants’ early understandings of effective interprofessional teamwork. The six dimensions are:

- Shared commitment
- Shared identity
- Clear team goals
- Clear roles and responsibilities
- Interdependence between team members
- Integration between work practices

Most dimensions were exemplified by some participants in more sophisticated understandings and conceptualisations, compared to other participants. Therefore, during the inductive analysis phase, different stages of learning in relation to each dimension inferred from the data were summarised. This resulted in two categories which we termed as Stage 1 (‘nascent’) learning, and Stage 2 (‘emerging’) learning. Samples of the data for these Stages are presented under each dimension in the following section with sample illustrative quotations and graphics from participants. Where both the participant quotation and drawing linked to the Stage learning, these are presented together. We have chosen to include a drawing for the most sophisticated stage of learning related to each dimension. The number in brackets (e.g. 205) indicates the unique identifier for that participant, and where this identifier number also refers to the drawing (Figure), this is indicated. One figure from Stage 1 has been redrawn (Created with BioRender.com), with a copy of one original figure to illustrate each dimension provided as Supplementary additional files.

### Shared commitment

Shared Commitment refers to the “psychological attachment that healthcare professionals feel towards their team” 10(p422). Some participants saw themselves as being committed to work with other team members, rather than articulating an attachment to the team itself. Sometimes this commitment was depicted in graphics emphasising the co-location of team members. That is, teamwork was viewed as a co-located activity, as described by the following participant:

*Got the client lying on the bed, and I’ve got an OT [occupational therapist], a physio, a psychologist, a nurse, and a doctor, all doing their thing in the same kind of area. (205)*

Beyond this notion of everyone engaged in their separate professional activities (“doing their thing”) while co-located, other participants linked co-location to limited interactive teamwork more explicitly.

Given these participants focus on the individual being committed to working with team members, for Shared Commitment, Stage 1 learning was defined as an “Individuals in the team committed to working together”.

**Fig. 1:**
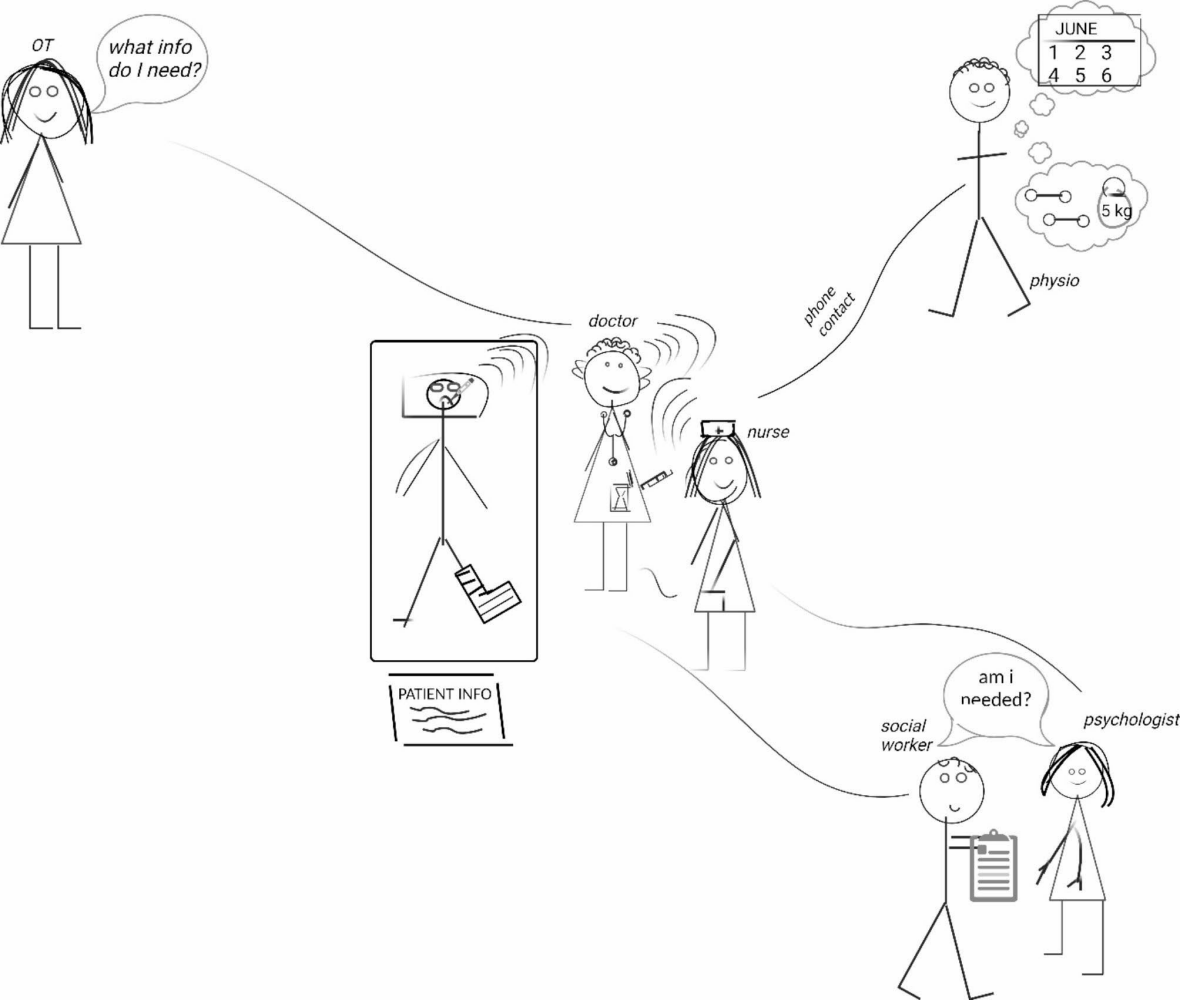
The doctor and nurse lead care but call on the expertise of other health professionals as needed (Shared Commitment Stage 2)

Stage 2 learning for Shared Commitment was shown in graphics by participants who demonstrated a shared commitment to how they would work together. For example, each team member was shown as being committed to engage in self-reflective activities or to communicate with each other:

*I think reflection is pretty important so that you can constantly improve. If something goes wrong, you shouldn’t just do the same thing again and hope it goes well* … *Even if everything goes right, there is still room for improvement.* (683: including Fig. 1 below, created with BioRender.com, and Additional file 1: Supplemental Fig. 1.)

These representations were evidence of a more sophisticated understanding of shared commitment beyond co-location. This Stage 2 was summarised as a “Team shares a commitment to working together effectively”.

### Shared identity

Shared Identity refers to the “collection of meanings attached to their team by healthcare professionals” 10(p422). Given participants were only in first year of university, they were beginning to identify with a profession.

Stage 1 learning was shown by several participants when they represented elements of professional identity. For some, this included depiction of the roles of others, as seen here:

*So, what I may do is different from what a doctor would do, but basically at the end of the day I’m giving the doctor information to help them make the decision on what the diagnosis is… At the end of the day, it all comes back to the common goal of making the correct diagnosis.* (464)

For other participants, the element of professional identity depicted was a sense of belonging to their own distinct professional group. The need to set aside professional differences during patient care was highlighted. This Stage 1 learning for Shared Identity was seen as “Individuals identify with their profession”.

Stage 2 learning for Shared Identity was seen when participants identified as a team, usually in respect of interpersonal relationships. For them, this shared identity was a sense of belonging and being part of the team:

*It’s about thinking about who else is there and what you should be doing and what your role is. Yeah, I guess like bouncing off each other, not just doing things by yourself and focusing on what’s going to affect you. You should see yourself as a team and the outcomes are the responsibility of everyone, not just one person or another.* (683, including Additional file 2: Supplemental Fig. 2.)

In summary, Stage 2 learning for Shared Identity was captured as: “Team identity is tied to interpersonal relationships”.

### Clear team goals

Xyrichis et al. 10(p422) refer to the dimension of Clear Team Goals as “the explicit articulation of the purpose and ambition of the interprofessional team”.

Stage 1 learning was evident when participants described team goals via characteristics such as being inclusive, having diversity, being reflective, and having an ability to stay on task. An example of these team characteristics is depicted below:

*They’re all doing their bit to help the patient … And these little platforms that they’re standing on are all the different things you need … Qualities you need in order to have a successful environment in a team.* (565).

Openness was another attribute that was noted as important to interprofessional teamwork.

Stage 1 learning was illustrated through a focus on desirable characteristics of the team and was therefore labelled: “Team goal is to have desirable characteristics”.

Stage 2 learning showed a more nuanced thinking about team goals. Participants emphasised the team goal was about helping the patient, achieved through activities such as discussing, being sociable and staying abreast of patient information as depicted by the following example:

*But it’s always the patient because you’ve gone into this health profession to help people […] so yeah, Bob is more important than everyone else […] doctors can’t necessarily give the best plan to someone with muscle atrophy for example, but a physiotherapist can.* (832, including Additional file 3: Supplemental Fig. 3.)

Stage 2 learning was illustrated by a focus on the patient and the purpose of the team was the patient. As a result, Stage 2 learning was labelled “Team purpose is patient centred: focus on individual accountabilities”.

### Clear roles and responsibilities

Xyrichis et al. 10(p422) defined Clear Roles and Responsibilities as “the differentiation of health care professional jurisdiction among the interprofessional team members”.

In Stage 1 learning, participants viewed the responsibilities of team members in terms of knowing the professional roles of other members. For example:

*And probably the nametags, as everyone should know who everyone is and what they do. Because if you don’t know what a person sitting next to you actually does then you’re not going to value their role and are not going to know how to use them to help you do the best you can do.* (313)

Other participants demonstrated understanding that each profession has different responsibilities, which they linked to the knowledge base of the profession. As well as different responsibilities, some participants discussed the professions as having a different relationship with patients.

Many participants had a simplistic view of how team communication is managed. For example, one participant who had drawn each team member holding a black notebook described this as symbolic of everyone “being on the same page”. Linear communication, with everyone taking a turn to speak was also a commonly held view. Some participants discussed the notion that professions vary based on individual competence in specific areas with little notion of shared competence.

Only one participant noted that nuance exists within each profession based on context. Medicine was used as an example to contrast the difference between different medical specialities:

*When someone says ‘doctor’, they might immediately think about their GP, but a general practitioner is different to a doctor. A GP recommends them to specialists. They have a little bit of a niche for example a general emergency room doctor, they might have some sort of niche they’ve gone into.* (832)

The link between role clarity and the safety/quality of care was noted by some. Others focused on leadership within the interprofessional team.

In summary, Stage 1 learning highlighted that study participants saw the importance of different professions holding different responsibilities, linked with their knowledge base. Stage 1 learning was labelled: “Each team member has a professional knowledge base”.

Stage 2 learning focused on the different responsibilities held by different professions. Limited examples of this stage were observed, such as:

*If the family needs support, then in this situation a psychologist and occupational therapy might be more important than a nutritionist, or if we’re talking about the patient needing to get better, then a pharmacist might be much more important because they prescribe anti-psychotic medication.* (855: including Additional file 4: Supplemental Fig. 4)

Stage 2 learning was labelled: “Professions carry different responsibilities”.

### Interdependence between team members

Interdependence between Team Members was defined as “extent to which the outcome of an interprofessional interaction depends on the decisions and choices of all team members” 10(p422).

None of the study participants described aspects of team interdependence but focused on being dependent on each other within the team. In Stage 1 learning, team members relied on information from each other:

*What it’s supposed to show is the meeting between all the health professionals and the patient making sure that everyone knows what’s going on. So just making everyone aware and bringing everyone as part of that patient’s care in together.* (321)

Another way dependence was depicted was in connection with patient referral.

In summary, Stage 1 learning for Interdependence was labelled “Team members depend on each other”.

In Stage 2 learning, participants showed an understanding that information from one team member could affect the decisions of another team member, and thus the patient:

*I know when I was seeing a psychologist and a psychiatrist, they were in contact, just to go over what was happening with my medication, how I was doing in school, all that kind of stuff.* (461, including Additional file 5: Supplemental Fig. 5.)

In summary, some participants illustrated the impact of one team member’s care of patient on another team member’s care and therefore Stage 2 learning was labelled: “Team members affect each other”.

### Integration between work practices

Integration between Work Practices was defined by Xyrichis et al. 10(p422) as “alignment of professional practice towards a whole product to which healthcare professionals contribute”. The product being “improved safety, quality, efficiency or care planning”.

In Stage 1 learning, participants remained focused on the individual to improve patient care, rather than the interprofessional team as a collective. Instead of aligning together for professional practice, participants saw team members working in parallel. Conflict was discussed in relation to scheduling, misunderstanding leading to frustration, and having different opinions:

*It is important in healthcare professionals to talk about their opinions and any values that can create conflict … Each other’s different values and opinions can cause conflict in a team. But because there is communication, they can work it out.* (609, including Additional file 6: Supplemental Fig. 6.)

Rather than integration of work practices, most participants focused on communication—active listening, asking questions—as a key to patient safety and quality.

In Stage 1 learning, participants referred to being aware of the work of others and ensuring that the work of others was communicated to each other. Stage 1 learning was labelled “Team members are aware of work of individual members”.

No Stage 2 learning was identifiable in the data for the dimension of Integration between Work Practices, so no participant quotations or graphics are displayed.

### Stages of learning

The stages of learning inferred from the data were aligned with each dimension of interprofessional activity, based on Xyrichis et al. [10]. All participants showed Stage 1 learning and many showed Stage 2 learning in some dimensions. Table 2 illustrates a summary of the participants’ learning, for each dimension of interprofessional activity [10], illustrated by the data. In the table, themes linked to each stage of learning and an indicative quotation are included.

**Table 2:** Stages of interprofessional learning for health professionals

Dimension of Interprofessional Activity and Learning Stage	Theme	Indicative Quotation (abbreviated)
**Shared Commitment**^ (dimension defined by Xyrichis et al., 2018 p.422)		
Stage 1* (nascent learning)	Individuals in the team committed to working together	They can all work in and around and amongst each other. (565)
Stage 2** (emerging learning)	Team shares a commitment to working effectively together	The doctor and nurse. They’ll likely be seeing the patient together, so they’re talking openly and listening. (462)
**Shared identity**^		
Stage 1	Individuals identify with their profession	They all have different expertise so they’re trying to balance, like, trying to do what they do best, but also bearing in mind there is four other health professionals helping as well. (205)
Stage 2	Team identity is tied to interpersonal relationships	Everyone’s sort of talking and everyone knows that they’re part of the team, and they’re not being left out or thought of being less equal to others. (301)
**Clear Team Goals**^		
Stage 1	Team goal is to have desirable characteristics	Everyone needs to be open minded … open to new perspectives, open to new ways of learning, open to new goals. (682)
Stage 2	Team purpose is to be patient/client centred; focus on individual accountabilities	Discussion is important because it allows areas to be thoroughly checked, which would be there is a better quality of care been given to patients. I think it increases safety … Also making an effort to stay up-to-date and collaborate. (461)
**Clear Roles and Responsibilities**^		
Stage 1	Each team member has a professional knowledge base	OT and physio share similar roles.… What a nurse does is very different to a surgeon, I think, but yeah, their relationship to the patient would be different as well. (188)
Stage 2	Professions carry different responsibilities	So it’s understanding your role and when it’s more important that you should push other people a certain way and say, ‘I can help with this’, and when it’s important you have to take a step back and leave it to others. (462)
**Interdependence between team members**^		
Stage 1	Team members depend on each other	I think it’s important that the OT tells the psychologist about that [patient management] instead of just assuming ‘that happened in my care, that’s not a problem. Keeping others informed. (461)
Stage 2	Team members affect each other	Because the dosage of medicine can have an impact on patients, so the communication between the two [pharmacist and doctor] is important as well, to talk about what’s right medicine to give. (609)
**Integration between work practices**^		
Stage 1	Team members are aware of work of individual members	There was some person adding that on record to, like the patient’s file, so that if another person who wasn’t present in the meeting saw the nurse attending that patient, they wouldn’t also attend. (301)
Stage 2	Not identified in the data	

^dimension defined by Xyrichis et al., 2018 p.422

*nascent learning

**emerging learning

## Discussion

An important education goal is that health professional students learn the competencies for effective interprofessional collaborative practice through carefully designed interprofessional education (IPE) experiences. Capturing participants’ understandings of interprofessional teamwork early in their degree studies provided us with insights into how our participants were learning about interprofessional teamwork.

The findings of this study provided examples that illustrated two stages of learning from participants during their initial encounters with IPE. Ultimately, we see the end point of learning as when teams demonstrate the dimensions of interprofessional activity detailed by Xyrichis et al. [10]. This is not to suggest that interprofessional teams or individuals stop learning, but rather that the end point is what is demonstrated by high functioning interprofessional teams. It is not surprising that we did not identify data linked with the end point of learning, given all participants were just beginning a health professions degree, and many recently completed secondary school education.

Evidence of Stage 1 learning across the six dimensions of the framework indicated most students had a nascent understanding of interprofessional teamwork, focused on an individualistic rather than a collective view. Student views aligned with what Katzenbach and Smith [19] described as a working group. A working group’s performance is a function of what its members do as individuals. In contrast, a team’s performance includes both individual results and a collective work-product; a product that two or more members work on together that reflects the joint contribution of the team members [19]. Study participants understood interprofessional teamwork to be focused on individual accountability and no indication of mutual accountability. Sharing information, having a basic understanding of each profession’s role and responsibilities, and sharing the goal of providing care for the patient were the essential elements of interprofessional teamwork identified by the participants. Perhaps this is not surprising given the focus of initial IPE is frequently on learning about the roles of other healthcare professions, the importance of patient centred care and interprofessional communication [20, 21].

According to Katzenbach and Smith [19] teams rely on more than sharing information, group discussion, debate, and decision making. Effective teams require a shared commitment to how they will work together to accomplish their goals. Team members must agree on who will do particular tasks, how schedules are set and adhered to, what competencies need to be developed, and how the group will make and modify decisions. This nuanced understanding of teamwork was absent from this group of first year students. Some participants identified elements of effective teamwork such as each profession being responsible for specific tasks, but they understood the enactment of teamwork to rely on the co-location of team members, face-to-face communication (joint patient sessions and meetings) and shared patient information.

There was one dimension for which students remained at Stage 1 learning and had not yet shown Stage 2 learning. There was no data related to Integration between Work Practices. This is of particular interest because IPE has historically focused on foundational competencies (e.g. role clarification, team communication) over the development of an interprofessional identity [22] or educating students about how interprofessional team members integrate their practice to achieve collective work-products [23]. The study participants viewed different health professions as working in largely separate professional communities.

Participants had yet to develop a more nuanced understanding of how responsibility for decision making in healthcare differs with patient and context. Participants tended to believe that awareness of the work of others and communicating one’s own work was sufficient. This restricted their understandings about the interdependence of interprofessional teamwork. Shared decision-making by the interprofessional team [24] was yet to enter participant thinking.

There was little evidence that participants understood ‘power’ within a healthcare team [25]. Participant thinking was constrained to stereotypes of hierarchy (nurses and doctors) rather than how power might shift in an interprofessional team and where there might be overlap in roles. Furthermore, almost all participants viewed each health profession as homogenous; all members of each profession had the same role and competence. These simplistic conceptualisations of professions and teams meant the complexity of power, status and context remained unexplored.

These findings have implications for pedagogical strategies and the way IPE experiences are designed. It appears important to design strategies that perturb ‘myths’ about the stability of healthcare teams and roles [5]. Further, health professionals need to engage in interprofessional teamwork both synchronous and asynchronously where many healthcare providers rotate in and out of the team on an ad hoc basis [26]. Pedagogical strategies that disrupt stereotypical views of health professional roles as discrete and acontextual are necessary, along with strategies and tools for managing the variability in healthcare.

Our findings suggest pedagogical strategies should introduce more complex, and sometimes more troubling concepts, such as team conflict during initial IPE. Learning activities could deliberately present phenomena of dysfunction or imperfection, alongside functioning teams. Authentic, not always idealistic, examples of interprofessional activities will help students learn a more balanced picture of the complexity and challenges of interprofessional teamwork. Our findings suggest this learning needs to happen during early exposure to IPE. Ultimately, such early IPE experiences will help students learn contextual ‘collective competence’ [5] in interprofessional teams.

### Limitations

There are limitations to our findings. The participants were from a range of healthcare courses but with small numbers. There was also a higher representation of psychology students, due to those students being required to demonstrate contribution to research during university studies. This may limit generalisations to other healthcare students. Methodologically, cross-comparative analysis across the two data sets (graphics and interviews) allows identification of commonalities but no contradictions were identified in the analysis, suggesting that the main strength of the graphic elicitation methods was as a stimulus for interview. Further research may indicate more stages for learning about interprofessional teamwork than the two stages we outlined.

## Conclusion

To prepare students for the reality of interprofessional teamwork, we recommend educators pay attention to stages of student learning when designing IPE experiences. The interprofessional activity framework utilised in this study provides one option for planning these learning stages. Some of the more challenging aspects identified in our study—learning about integration as a team—need particular focus so students develop more sophisticated understandings. For educators, one implication of our study is that early exposure to interprofessional teams with greater diversity of functioning is important to assist students through stages of learning. Whilst further research is needed, the two stages of learning we have outlined assists building knowledge about how to facilitate student learning about the dimensions of interprofessional teamwork, to advance healthcare systems.

### Electronic supplementary material

Below is the link to the electronic supplementary material.

Supplementary Material 1

Supplementary Material 2

Supplementary Material 3

Supplementary Material 4

Supplementary Material 5

Supplementary Material 6

## Data Availability

The datasets analysed during the current study are not publicly available due to university confidentiality arrangements. Please contact the corresponding author regarding data access.

## Declarations

### Abbreviations

NA.
