# Supplementary material for: Early stages of learning in interprofessional education: stepping towards collective competence for healthcare teams
Source: BMC Med Educ. 2023 Sep 22;23:694. doi: 10.1186/s12909-023-04665-8 (PMC10517498; doi:10.1186/s12909-023-04665-8)

**Additional File 1**

Supplemental Figure 1: The doctor and nurse lead care but call on the expertise of other health professionals as needed (Shared Commitment Stage 2)


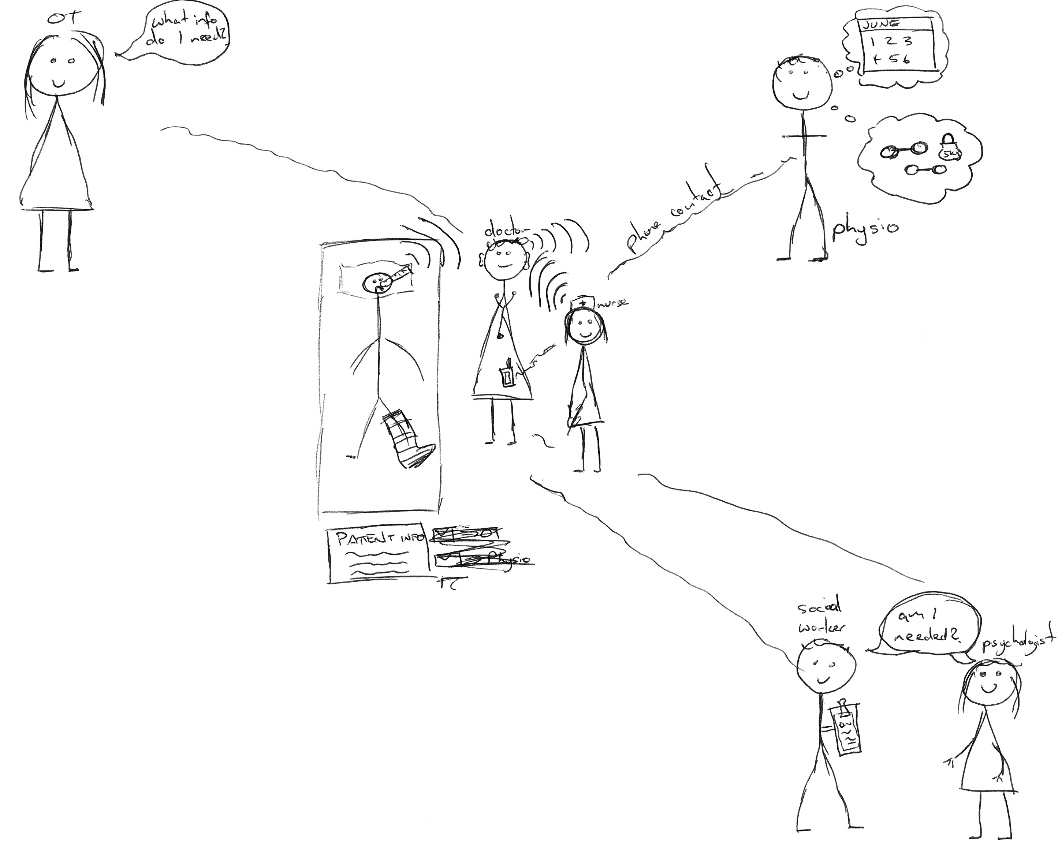

Supplement: Supplementary file 1 — Supplementary Material 1 [file 12909_2023_4665_MOESM1_ESM.docx]
