# Supplementary material for: Early stages of learning in interprofessional education: stepping towards collective competence for healthcare teams
Source: BMC Med Educ. 2023 Sep 22;23:694. doi: 10.1186/s12909-023-04665-8 (PMC10517498; doi:10.1186/s12909-023-04665-8)

**Additional file 2**

Supplemental Figure 2: Team meetings facilitate planning of care that is delivered as a team, followed by reflection on team outcomes (Shared Identity Stage 2)


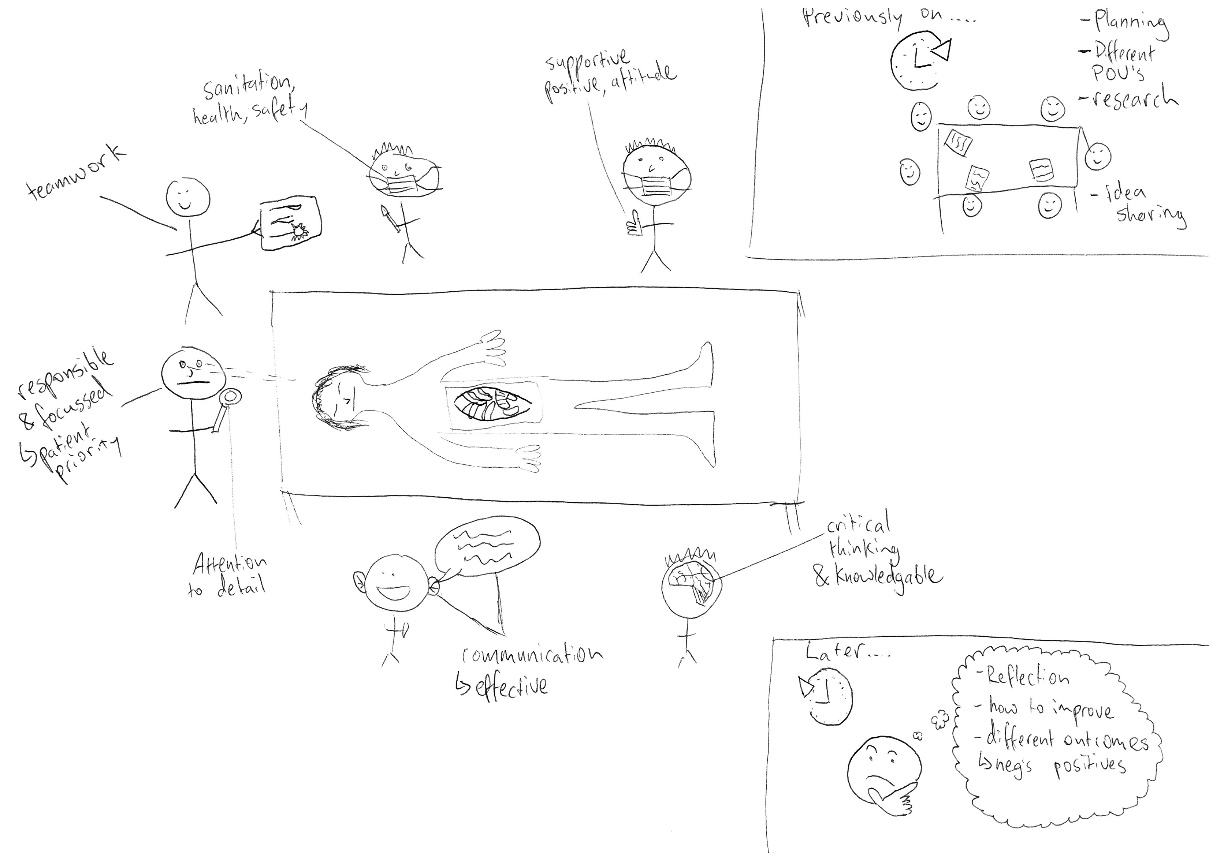

Supplement: Supplementary file 2 — Supplementary Material 2 [file 12909_2023_4665_MOESM2_ESM.docx]
