# Supplementary material for: Early stages of learning in interprofessional education: stepping towards collective competence for healthcare teams
Source: BMC Med Educ. 2023 Sep 22;23:694. doi: 10.1186/s12909-023-04665-8 (PMC10517498; doi:10.1186/s12909-023-04665-8)

Additional file 3

Supplemental Figure 3: The patient is referred to different health professionals throughout their recovery journey (Clear Team Goals Stage 2)


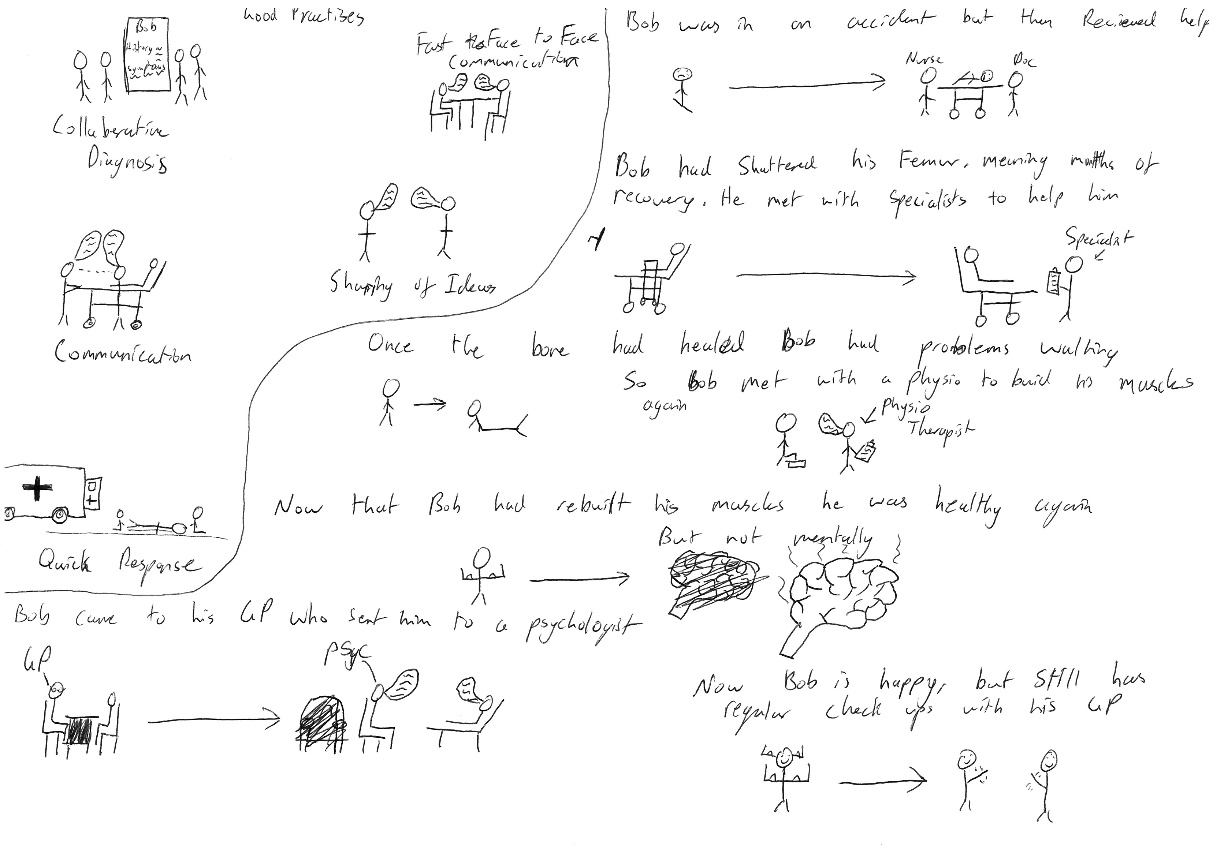

Supplement: Supplementary file 3 — Supplementary Material 3 [file 12909_2023_4665_MOESM3_ESM.docx]
