# Supplementary material for: Early stages of learning in interprofessional education: stepping towards collective competence for healthcare teams
Source: BMC Med Educ. 2023 Sep 22;23:694. doi: 10.1186/s12909-023-04665-8 (PMC10517498; doi:10.1186/s12909-023-04665-8)

**Additional file 4**

Supplemental Figure 4: The doctor leads the care of a patient with schizophrenia but needs to work together with other health professionals who make a specific contribution to care (Clear Roles and Responsibilities Stage 2)


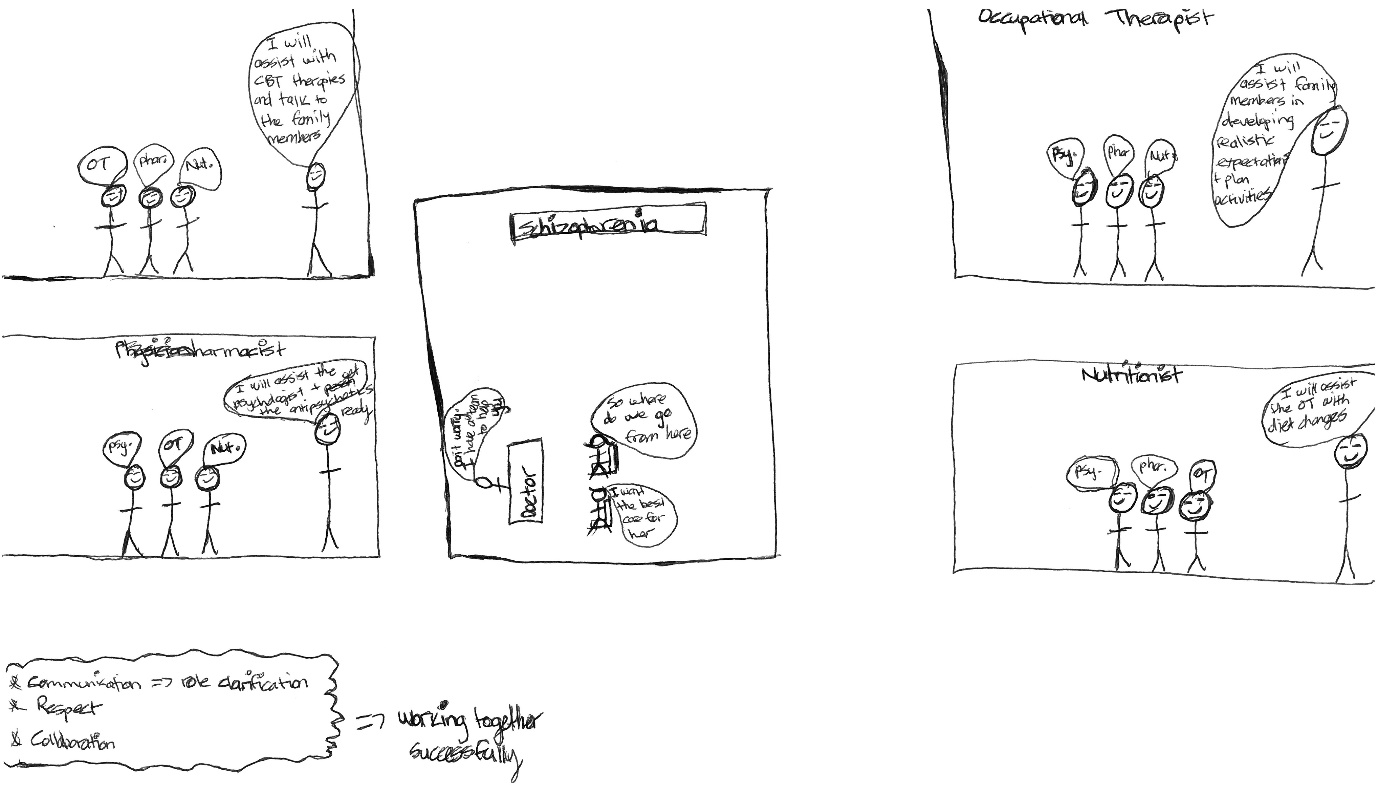

Supplement: Supplementary file 4 — Supplementary Material 4 [file 12909_2023_4665_MOESM4_ESM.docx]
