# Supplementary material for: Early stages of learning in interprofessional education: stepping towards collective competence for healthcare teams
Source: BMC Med Educ. 2023 Sep 22;23:694. doi: 10.1186/s12909-023-04665-8 (PMC10517498; doi:10.1186/s12909-023-04665-8)

**Additional file 5**

Supplemental Figure 5: Direct and indirect communication is critical to ensuring role understanding and coordinating care (Interdependence between Team Members Stage 2)


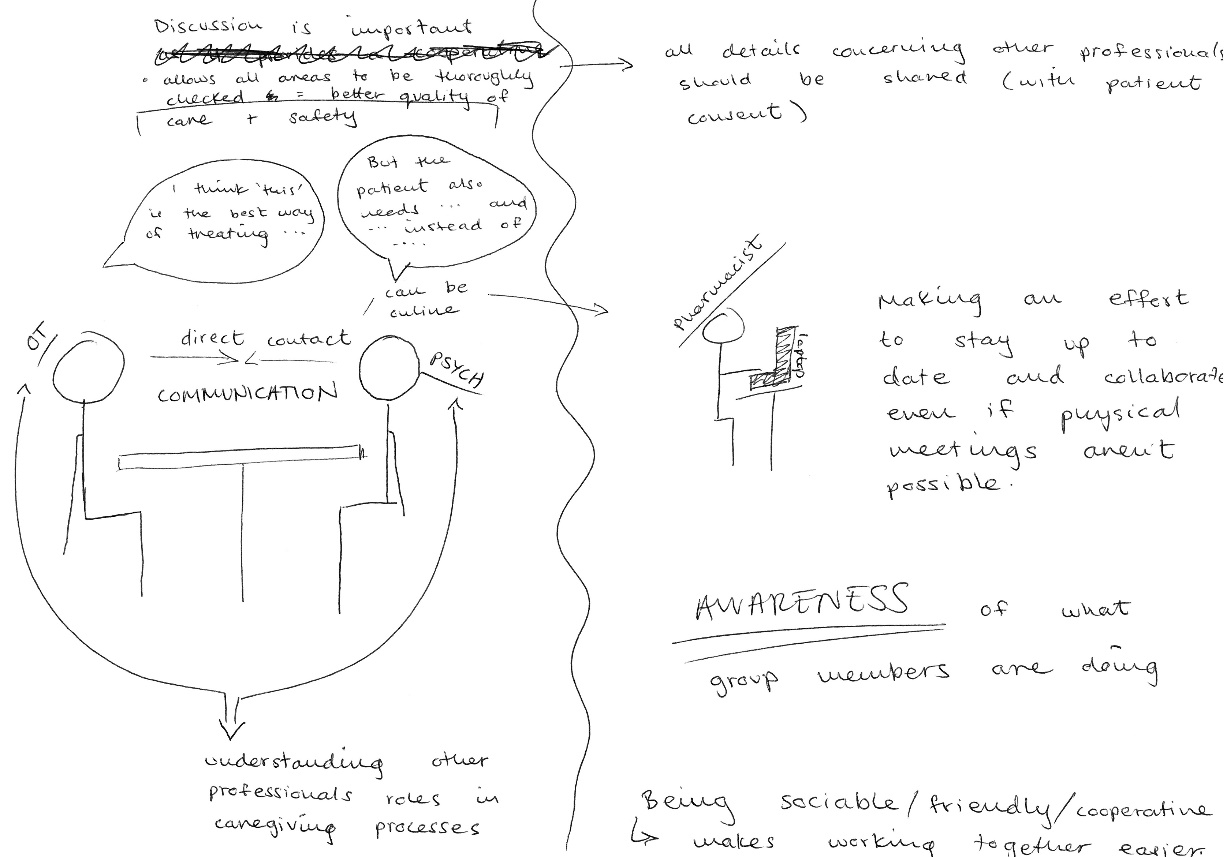

Supplement: Supplementary file 5 — Supplementary Material 5 [file 12909_2023_4665_MOESM5_ESM.docx]
