# Supplementary material for: Early stages of learning in interprofessional education: stepping towards collective competence for healthcare teams
Source: BMC Med Educ. 2023 Sep 22;23:694. doi: 10.1186/s12909-023-04665-8 (PMC10517498; doi:10.1186/s12909-023-04665-8)

**Additional file 6**

Supplemental Figure 6: Communication between health professionals can occur in a multiple of ways (Integration between Work Practices Stage 1)


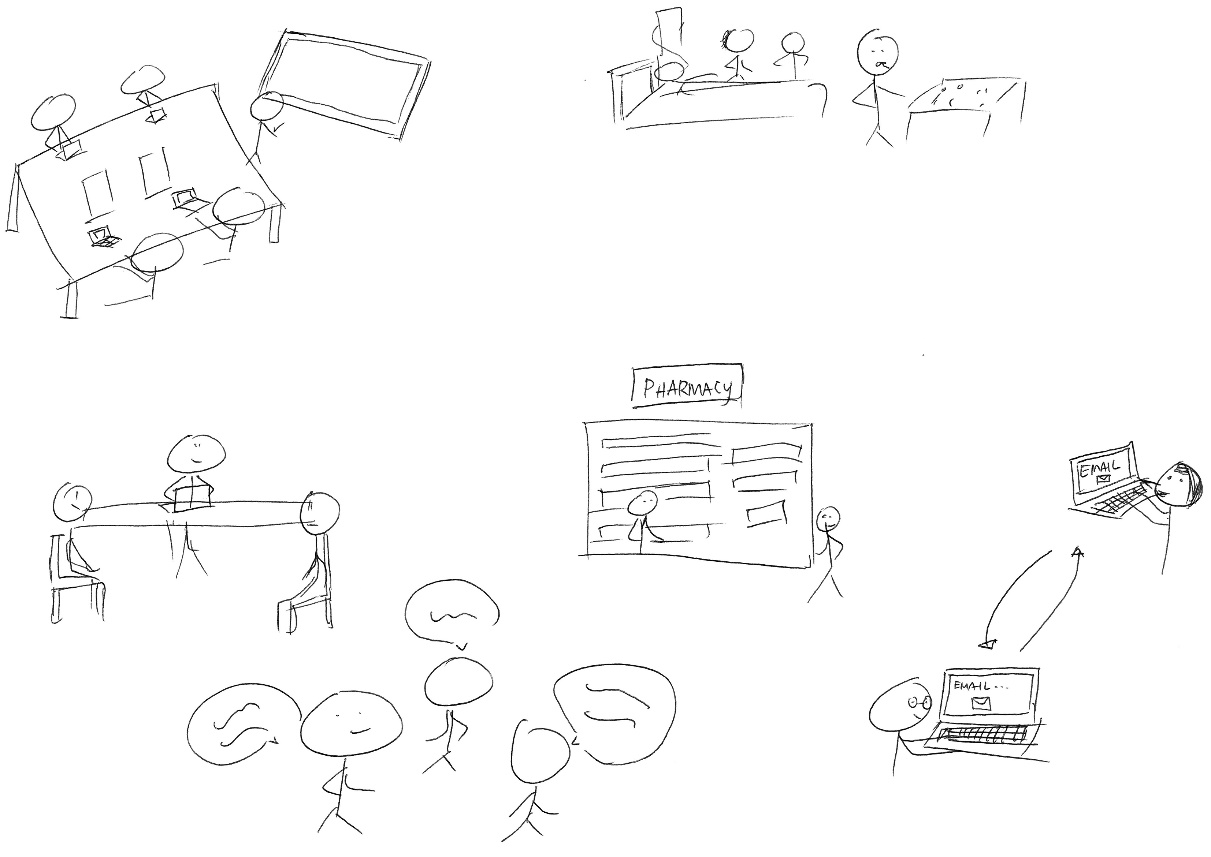

Supplement: Supplementary file 6 — Supplementary Material 6 [file 12909_2023_4665_MOESM6_ESM.docx]
